# Piperine Attenuates Cigarette Smoke-Induced Oxidative Stress, Lung Inflammation, and Epithelial–Mesenchymal Transition by Modulating the SIRT1/Nrf2 Axis

**DOI:** 10.3390/ijms232314722

**Published:** 2022-11-25

**Authors:** Pritam Saha, Sneha Durugkar, Siddhi Jain, P. A. Shantanu, Samir R. Panda, Aishwarya Jala, Sharad Gokhale, Pawan Sharma, V. G. M. Naidu

**Affiliations:** 1 Department of Pharmacology & Toxicology, National Institute of Pharmaceutical Education and Research Guwahati, Changsari, Guwahati 781101, Assam, India; 2 Department of Pharmaceutical Analysis, National Institute of Pharmaceutical Education and Research Guwahati, Changsari, Guwahati 781101, Assam, India; 3 Department of Civil Engineering, Indian Institute of Technology Guwahati, Guwahati 781039, Assam, India; 4 Division of Pulmonary, Allergy and Critical Care Medicine, Centre for Translational Medicine, Jane & Leonard Korman Respiratory Institute, Sidney Kimmel Medical College, Thomas Jefferson University, Philadelphia, PA 19107, USA

**Keywords:** inflammation, EMT, piperine, cigarette smoke, airway disease, antioxidant

## Abstract

Piperine (PIP) is a major phytoconstituent in black pepper which is responsible for various pharmacological actions such as anti-inflammatory, antioxidant, and antitumor activity. To investigate the effects and mechanisms of PIP on cigarette smoke (CS)-induced lung pathology using both in-vitro and in-vivo models. BEAS-2B and A549 cells were exposed to CS extract (CSE) for 48 h; BALB/c mice were exposed to CS (9 cigarettes/day, 4 days) to induce features of airway disease. PIP at doses of (0.25, 1.25, and 6.25 µM, in vitro; 1 and 10 mg/kg, in vivo, i.n) and DEX (1 µM, in vitro; 1 mg/kg, in vivo, i.n) were used to assess cytotoxicity, oxidative stress, epithelial–mesenchymal transition (EMT), Sirtuin1 (SIRT1), inflammation-related cellular signaling, and lung function. PIP treatment protects cells from CSE-induced lung epithelial cell death. PIP treatment restores the epithelial marker (*p* < 0.05) and decreases the mesenchymal, inflammatory markers (*p* < 0.05) in both in vitro and in vivo models. The PIP treatment improves the altered lung function (*p* < 0.05) in mice induced by CS exposure. Mechanistically, PIP treatment modulates SIRT1 thereby reducing the inflammatory markers such as IL-1β, IL-6 and TNF-α (*p* < 0.05) and enhancing the epigenetic marker HDAC2 (*p* < 0.05) and antioxidant marker Nrf2 (*p* < 0.05) expressions. Thus, PIP alleviates pulmonary inflammation by modulating the SIRT1-mediated inflammatory cascade, inhibits EMT, and activates Nrf2 signaling.

## 1. Introduction

Airway inflammation is one of the cardinal features of chronic pulmonary diseases such as chronic obstructive pulmonary disease (COPD) and asthma. COPD is a progressive and multifactorial disease initiated by various genetic and environmental risk factors. It comprises bronchial asthma, chronic bronchitis, and pulmonary emphysema. It is characterized by airway inflammation, airflow limitation, irreversible lung damage mainly in the alveoli, and gradual decline in lung function as the disease progresses [1]. According to WHO, approximately 3.17 million people suffer from chronic airway inflammatory diseases, of which 2.51 million patients die yearly [2]. Due to increased industrial activity, urbanization and climate change will aggravate chronic airway disease incidence and mortality rates. Several studies have also revealed that chronic ubiquitous air pollution exposure leads to COPD development and exacerbation due to enhanced oxidative stress and inflammatory cascades [1]. With the current incidence rate, chronic lung diseases are projected to be the third leading cause of mortality by 2030. [2]. Chronic inflammation remains the primary driver of various progressively irreversible physiological changes in normal lungs. These physiological changes include, but are not limited to, multiple structural abnormalities of the airways and damaged alveoli, such as excessive mucus production, constricted and damaged bronchoalveolar airways, and replacement of ciliated columnar epithelial cells with squamous cells. These changes limit the efficiency of normal lungs for gaseous exchange, which leads to extrapulmonary complications such as stroke and heart failure. Furthermore, chronic inflammation also induces epigenetic changes in the affected cells that cause airway hyperresponsiveness, a primary physiological trait observed in asthma and COPD. Persistent inflammation results from long-term exposure to various allergens, atmospheric contaminants such as smoke, pollens, particulate matter, etc., and exhaust gases released due to human activities [3]. 

Continuous exposure to cigarette smoke in both active and passive smokers induces changes that cause chronic inflammation, leading to dysregulation of Sirtuin1 (SIRT1) in COPD and asthma patients [4,5]. SIRT1 is a nicotinamide adenine dinucleotide (NAD^+^)-dependent histone deacetylase. It regulates the antioxidant and inflammatory pathways by modulating the expression of nuclear factor erythroid 2–related factor 2 (Nrf2) and nuclear factor kappa B (NF-κB). SIRT1 also holds epigenetic modification in inflammatory and antioxidant defense genes [6,7]. Prolonged inflammation and oxidative stress due to dysregulated SIRT1 epigenetic modifications activate epithelial–mesenchymal transition (EMT) [8]. The EMT process leads to a loss of polarity in epithelial cells, which results in the replacement of epithelial cells with mesenchymal cells. The hyperactivated EMT signaling gradually leads to fibrosis in the small airways, constricting and limiting lung function efficiency as the disease progresses over time [9]. 

The current clinically available therapy only provides symptomatic relief from COPD and asthma. The clinical management involves a range of bronchodilators, inhaled corticosteroids, and phosphodiesterase 3/4 inhibitors. However, these drugs are limited in their ability to address the root cause behind chronic pulmonary inflammation; as a result, drug tolerance and resistance is observed in advanced stages, where drug treatment fails to relieve chronic inflammation [10]. Thus, there is an unmet medical need to discover drugs that effectively inhibit oxidative stress, inflammation, reverse EMT, and epigenetic changes in exposed airways that could effectively treat chronic pulmonary inflammation [11,12]. 

Piperine (PIP), a major alkaloid found in black pepper (Piper nigrum, Piperaceae), has been reported to have antifibrotic [13], anti-inflammatory, and antioxidant properties [14,15,16]. Piperine has also been reported to alleviate lung injury in the LPS model via modulating NF-κB signaling pathways [17]. In addition, PIP has also been reported to inhibit TGF-β-mediated EMT changes in alveolar epithelial (A549) cells [18,19]. Due to the limitations of conventional therapy, many patients prefer an alternative system of medicine. Traditional medicine systems such as Ayurveda employ black pepper as a constituent in nasal formulation (nasyum) to treat patients with pulmonary inflammation. [20,21]. However, no scientific studies have reported the effects of PIP on cigarette smoke (CS)-induced pulmonary inflammation and EMT changes in lung cells after intranasal administration. Hence, the current study aims to find the protective effects of PIP on the modulation of the SIRT1/Nrf2 signaling axis in CS-induced lung pathology after its administration by the intranasal route in both in-vitro and in-vivo models. 

## 2. Results

### 2.1. CSE Exposure Results in the Loss of Cell Viability in Lung Epithelial Cells 

**Figure 1:**
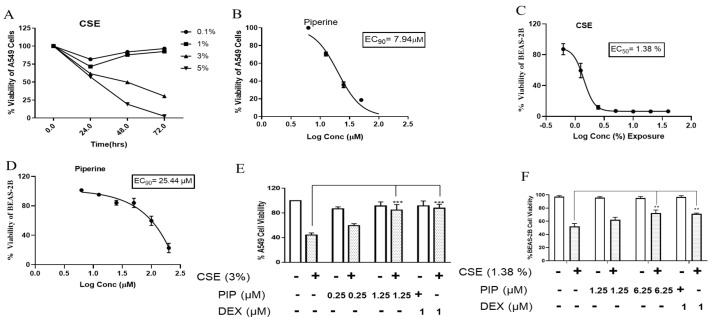
Cytotoxicity assessment of cigarette smoke and piperine by using MTT assay: (**A**) Dose-dependent cytotoxicity of cigarette smoke over the exposure of cigarette smoke at three different time points (24, 48, and 72 h) in A549 cells. (**B**) Represents % viability of A549 cell line exposed to different concentrations of PIP (0, 1.56, 3.12, 6.25, 12.5, 25, 50, and 100 µM). (**C**) Dose-dependent cytotoxicity was observed during the exposure of cigarette smoke at 48 h in BEAS-2B cells. (**D**) Represents % viability of BEAS-2B cell line exposed to different concentrations of piperine (0–200 µM). (**E**) Piperine treatment protects the CSE-induced cell death in A549 cells. (**F**) Piperine treatment protects the CSE-induced cell death in BEAS-2B cells. Values are expressed as mean ± SEM of triplicate experiment. *** *p* < 0.001, ** *p* < 0.01 vs. CSE exposed group. Data were analyzed using graph pad prism 7 software.

CS exposure is known to cause excessive apoptosis in exposed airway cells [22]. Thus, to mimic the cigarette smoke exposure in-vitro, cigarette smoke extract (CSE) was prepared by concentrating CS in PBS as described in the materials and methods. Nicotine is the major water-soluble component responsible for the physiological effects of addiction and toxicity. Nicotine content in CSE was found to be 0.367 μg/mL, estimated using HPLC (Supplementary Figure S1). CSE exposure significantly impacts cellular viability. The compounds with lung protective activity must be able to salvage cells from CSE-induced cytotoxicity [23]. A549, alveolar lung epithelial cells were exposed to various concentrations of CSE (0, 0.1, 1, 3, 5%, *v/v*) for 24, 48, and 72 h using an MTT assay. As illustrated in (Figure 1A), CSE treatment showed concentration and time-dependent reduction in cell viability. CSE at a concentration of 3% (*v/v*) exposed for 48 h caused a 50% reduction in cell viability. A similar trend was observed in BEAS-2B cells (Figure 1C). CSE exposure displayed a dose-dependent decrease in cell viability in BEAS-2B cells at 48 h, IC_50_ was 1.38% (*v/v*), as shown in (Figure 1C). Thus, 3% (*v/v*) and 1.38% (*v/v*) of CSE was exposed for 48 h in A549 and BEAS-2B cells, respectively, to find the effect of PIP for lung protective activities in all the following experiments.

### 2.2. Piperine Protects the CSE-Induced Cell Death in Lung Epithelial Cells

We used an MTT assay in A549 and BEAS-2B cells to estimate PIP’s protective doses. Increasing concentrations of PIP ranging from 0, 1.56, 3.12, 6.25, 12.5, 25, 50, 100 to 200 µM were treated to A549 and BEAS-2B cells for 48 h. The EC_90_ was estimated to gauge the maximum PIP concentration that can be used to explore the protective effects without significantly affecting the cell viability. The PIP EC_90_ was 7.94 µM in the case of A549 cells (Figure 1B) and 25.44 µM in BEAS-2B cells (Figure 1D). To evaluate the protective effect of PIP on CSE-induced epithelial cell death [23] we exposed the cells to 3% (*v/v*) CSE in A549 cells and 1.3% (*v/v*) in BEAS-2B cells, followed by a post-1 h treatment with PIP (at a concentration of 0.25 and 1.25 µM for A549 cells and 1.25 and 6.25 µM for BEAS-2B cells) for 48 h in both the cells. Dexamethasone (DEX) was used as a positive control because it is considered to be the first line therapy for the treatment of lung inflammation [24]. Higher concentrations of PIP (1.25 µM in A549 and 6.25 µM in BEAS-2B cells) displayed a protective effect against CSE-induced cytotoxicity (Figure 1E,F, *p* < 0.01 vs. CSE group for both cells). Thus, two concentrations of PIP, 0.25 and 1.25 µM in A549 and 1.25 and 6.25 µM in BEAS-2B cells, were chosen to explore the molecular mechanisms in which PIP is protecting the lung epithelial cells from CS-induced cytotoxicity. 

### 2.3. Piperine Curtails Oxidative Stress and Inflammation by Upregulating SIRT1, Nrf2 and Inhibiting NF-κB Translocation in Lung Epithelial Cells 

**Figure 2:**
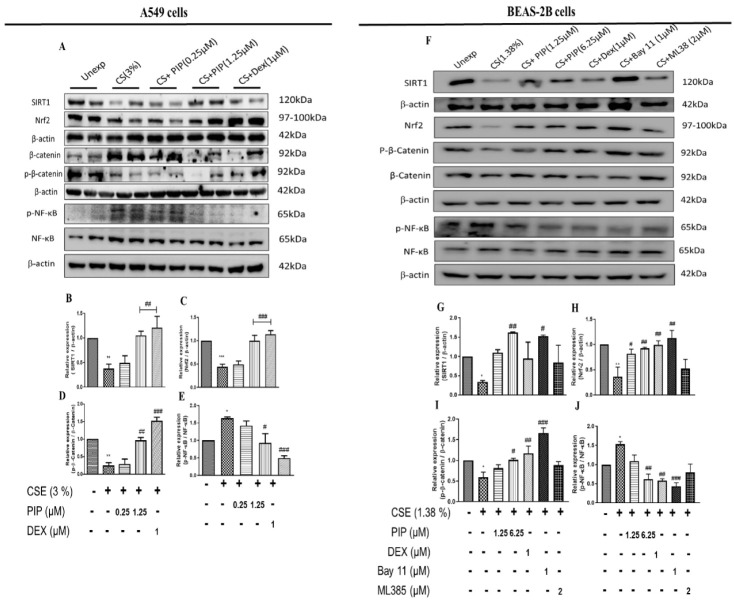
Effect of piperine treatment on expression of oxidative stress, antioxidant, Wnt, and SIRT1 signaling in CSE exposed airway cells: (**A**) Immunoblot images represented the expressions of SIRT1, Nrf2, p-β-Catenin/Total β-Catenin, and p-NF-κB/Total NF-κB in A549 cells. Bar graphs represented densitometry analyses of (**B**) SIRT1, (**C**) Nrf2, (**D**) p-β-Catenin/Total β-Catenin and (**E**) p-NF-κB/Total NF-κB. (**F**) Immunoblot images represented the expressions of SIRT1, Nrf2, p-β-Catenin/Total β-Catenin, and p-NF-κB/Total NF-κB in BEAS-2B cells. Bar graphs represented densitometry analyses of (**G**) SIRT1, (**H**) Nrf2, (**I**) p-β-Catenin/Total β-Catenin and (**J**) p-NF-κB/Total NF-κB. The values are expressed as mean ± SEM (*n* = 3–4). * *p* < 0.05, ** *p* < 0.01 and *** *p* < 0.001 vs. Unexposed, # *p* < 0.05, ## *p* < 0.01 and ### *p* <0.001 vs. CSE. For A549 cells: CSE: cigarette smoke extract-treated (3%), CS + PIP (0.25): cigarette smoke extract + 0.25 µM PIP, CS + PIP (1.25): cigarette smoke extract + 1.25 µM PIP, CS + DEX (1): cigarette smoke extract + 1 µM DEX. For BEAS-2B cells: CSE: cigarette smoke extract-treated (1.38%), CS + PIP (1.25): cigarette smoke extract + 1.25 µM PIP, CS + PIP (6.25): cigarette smoke extract + 6.25 µM PIP, CS + DEX (1): cigarette smoke extract + 1 µM DEX, CS + Bay 11 (1): cigarette smoke extract + 1 µM Bay 11, CS + ML385 (2): cigarette smoke extract + 2 µM ML-385.

**Figure 3:**
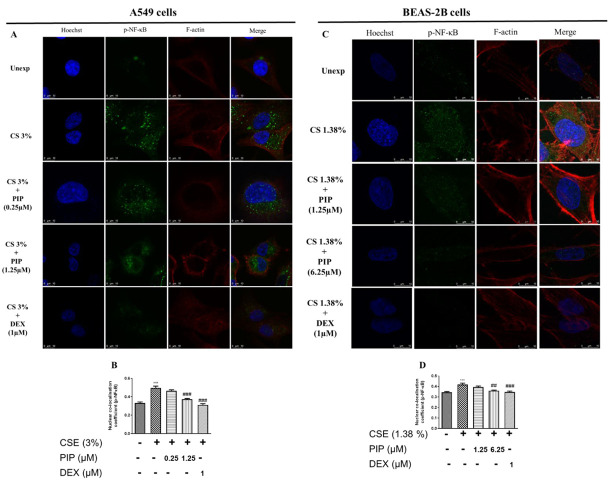
Effect of piperine treatment on expression of NF-κB signaling in CSE exposed airway cells. (**A**) Immunofluorescence staining assay representing an expression of p-NF-κB in A549 cells. Bar graphs representing a mean fluorescence intensity of (**B**) p-NF-κB. (**C**) Immunofluorescence staining assay representing an expression of p-NF-κB in BEAS-2B cells. Bar graphs representing a mean fluorescence intensity of (**D**) p-NF-κB. The values are expressed as mean ± SEM (*n* = 3). Blue colour indicates nuclei, green colour represents p-NF-κB protein expression, and red colour represents F-actin labelled with phalloidin red. *** *p* < 0.001 vs. Unexposed, ## *p* < 0.01 and ### *p* <0.001 vs. CSE. For A549 cells: CSE: cigarette smoke extract-treated (3%), CS + PIP (0.25): cigarette smoke extract + 0.25 µM PIP, CS + PIP (1.25): cigarette smoke extract + 1.25 µM PIP, CS + DEX (1): cigarette smoke extract + 1µM DEX. For BEAS-2B cells: CSE: cigarette smoke extract-treated (1.38%), CS + PIP (1.25): cigarette smoke extract + 1.25 µM PIP, CS + PIP (6.25): cigarette smoke extract + 6.25 µM PIP, CS + DEX (1): cigarette smoke extract + 1 µM DEX.

SIRT1 mediates the cellular defense against potential immunogens by regulating the expression of NF-κB and Nrf2 through epigenetic modifications [25]. CSE exposure (3% (*v/v*), and 1.38% (*v/v*), in A549 and BEAS-2B, respectively) resulted in oxidative stress and inflammation, as indicated by the increased NF-κB and reduced Nrf2 levels. The sustained inflammatory condition downregulated SIRT1 expression altering cellular epigenetics to maintain an inflamed state due to CSE exposure when compared to unexposed cells (Figure 2A,B, *p* < 0.01; Figure 2F,G, *p* < 0.05 and Figure 3A–D, *p* < 0.001). The treatment with PIP (1.25 µM in A549 and 6.25 µM in BEAS-2B) reversed CSE-induced changes by enhancing the Nrf2, inhibiting NF-κB, and thereby increasing SIRT1 expression levels when compared to the CSE-exposed group (Figure 2A–C, *p* < 0.01; Figure 2F,G, *p* < 0.05 and Figure 3A–D, *p* < 0.01). The effect of PIP was comparable to the standard DEX group (Figure 2A, B, *p* < 0.01, Figure 2F,G, *p* < 0.001 and Figure 3A–D, *p* < 0.01). We have also measured the expression of p-NF-κB/ Total NF-κB against CSE exposure in both cells. We found increased expression of p-NF-κB/ Total NF-κB (Figure 2A,E,F,J, *p* < 0.05), indicating the activation of NF-κB signaling inflammatory cascade. However, PIP treatment (1.25 µM in A549 cells; 6.25 µM in BEAS-2B cells) resulted in a decreased expression of p-NF-κB/Total NF-κB compared with the CSE- group (Figure 2A,E,F,J, *p* < 0.01); as a result, it diminished the p-NF-κB-induced inflammation via a decrease in the release of inflammatory mediators. The effect was similar to standard DEX (1 µM) treatment with p-NF-κB/ Total NF-κB compared with the CSE group (Figure 2A,E,F,J, *p* < 0.001). In addition, we also compared the effect of PIP against standard NF-κB and Nrf2 inhibitors, Bay-11 (1 µM) and ML-385 (2 µM) respectively, in CSE- (1.38% *v/v*) exposed BEAS-2B cells (Figure 2F,G, *p* < 0.05). We observed that at a high concentration of PIP (6.25 µM) and a standard NF-κB inhibitor, Bay-11 (1 µM) treatment increased SIRT1 expression when compared to the CSE group (Figure 2F,G, *p* < 0.05), indicating that PIP treatment modulates SIRT1 signaling as a result, decreasing NF-κB expression. Standard Nrf-2 inhibitor, ML-385 (2 µM), treatment showed no significant changes in the expression of SIRT1 compared with the CSE-exposed group, worsening the disease condition (Figure 2F,G), suggesting the need to regulate SIRT1 signaling to regulate Nrf2 levels. Moreover, the effect of PIP treatment on CSE-exposed (A549 and BEAS-2B) cells were similar to the standard Bay-11 (NF-kB inhibitor). The anti-inflammatory effects of PIP were due to its inhibition of expression and the nuclear translocation of p-NF-kB (Figure 3A–D, *p* < 0.001), which were elevated following CSE exposure, as suggested by immunoblotting and immunofluorescence experiments. The effects of PIP (6.25 µM) treatment were equipotent to DEX (1 µM) treatments that resulted in inhibition of NF-κB-mediated signaling by preventing nuclear translocation of p-NF-κB for both cells when compared with the CSE group (Figure 3A–D, *p* < 0.01). The PIP treatment also increased Nrf2 levels in CSE-exposed (A549 and BEAS-2B) cells (Figure 2A,C,F,H, *p* < 0.01), augmenting the anti-inflammatory effects of PIP.

### 2.4. Piperine Inhibits the Wnt Signaling in Lung Epithelial Cells 

Wnt remains the primary paracrine signaling mechanism that drives inflammation and transformation upon exposure to cigarette smoke [26]. The inhibition of the Wnt pathway curtails inflammation and has a cascading effect on inhibiting EMT in exposed airway cells. A549 and BEAS-2B cells following exposure to 3% *v/v* and 1.38% *v/v* CSE resulted in decreased p-β-catenin/β-catenin expression (Figure 2A,D,F,I, *p* < 0.05), indicating an activation of a Wnt-induced inflammatory cascade. PIP treatment at high concentration (1.25 µM in A549 cells; 6.25 µM in BEAS-2B cells) initiates degradation and inactivation of β-catenin, as evident from the increased expression of p-β-catenin/β-catenin when compared with the CSE- group (Figure 2A,D,F,I, *p* < 0.01); as a result, Wnt-induced inflammation is curtailed via a decrease in the release of inflammatory mediators. The effect was similar to a standard DEX (1 µM) treatment with p-β-catenin/β-catenin compared with the CSE group (Figure 2A,D,F,I, *p* < 0.001).

To confirm whether the NF-κB and Nrf2 inhibition of PIP (6.25 µM) translated to the inhibition of the Wnt signaling, we estimated the levels of p-β-catenin/β-catenin. We compared the effect of PIP against standard NF-κB and Nrf2 inhibitors, Bay-11 (1 µM) and ML-385 (2 µM) respectively, in CSE- (1.38% *v/v*) exposed BEAS-2B cells (Figure 2F,I, *p* < 0.05). We observed that at a high concentration of PIP (6.25 µM) and with a standard NF-κB inhibitor, Bay-11 (1 µM) treatment increased p-β-catenin/β-catenin compared with the CSE-exposed group (Figure 2F,I, *p* < 0.01), indicating a Wnt signaling inhibition via NF-κB inhibition. However, the treatment with ML-385 showed a similar effect with CS-exposed cells (Figure 2F,I), suggesting that Wnt signaling activation was independent of Nrf2 protein.

### 2.5. Piperine Diminishes Oxidative Stress, NF-κB, and Wnt Signaling Resulting in Inhibition of EMT in Lung Epithelial Cells 

**Figure 4:**
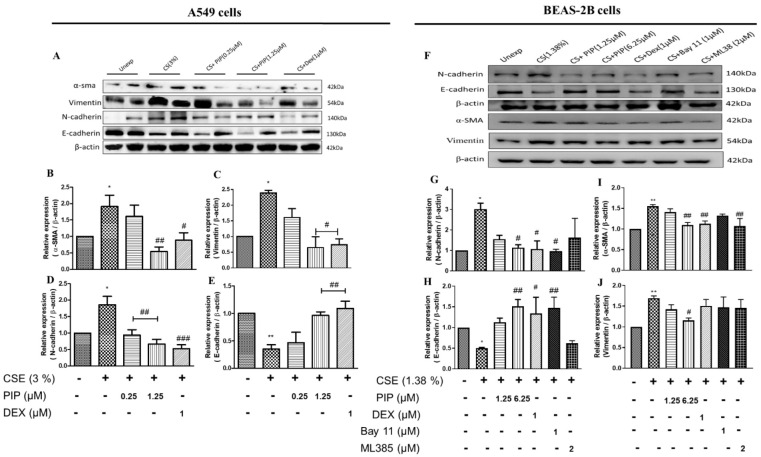
Effect of piperine treatment on expression of EMT signaling in CSE-exposed airway cells. (**A**) Immunoblot images representing the expressions of E-cadherin, N-cadherin, Vimentin, and α-SMA in A549 cells. Bar graphs representing densitometry analysis of (**B**) E-cadherin, (**C**) Vimentin, (**D**) E-cadherin and (**E**) α-SMA. (**F**) Immunoblot images representing the expressions of E-cadherin, N-cadherin, Vimentin and α-SMA in BEAS-2B cells. Bar graphs representing densitometry analysis of (**G**) N-cadherin, (**H**) E-cadherin. (**I**) α-SMA and (**J**) Vimentin. The values are expressed as mean ± SEM (*n* = 3). * *p* < 0.05 and ** *p* < 0.01, # *p* < 0.05, ## *p* < 0.01 and ### *p* < 0.001 vs. CSE. For A549 cells: CSE: cigarette smoke extract-treated (3%), CS + PIP (0.25): cigarette smoke extract + 0.25 µM PIP, CS + PIP (1.25): cigarette smoke extract + 1.25 µM PIP, CS + DEX (1): cigarette smoke extract + 1 µM DEX. For BEAS-2B cells: CSE: cigarette smoke extract-treated (1.38%), CS + PIP (1.25): cigarette smoke extract + 1.25 µM PIP, CS + PIP (6.25): cigarette smoke extract + 6.25 µM PIP, CS + DEX (1): cigarette smoke extract + 1 µM DEX, CS + Bay 11 (1): cigarette smoke extract + 1 µM Bay 11, CS + ML385 (2): cigarette smoke extract + 2 µM ML-385.

**Figure 5:**
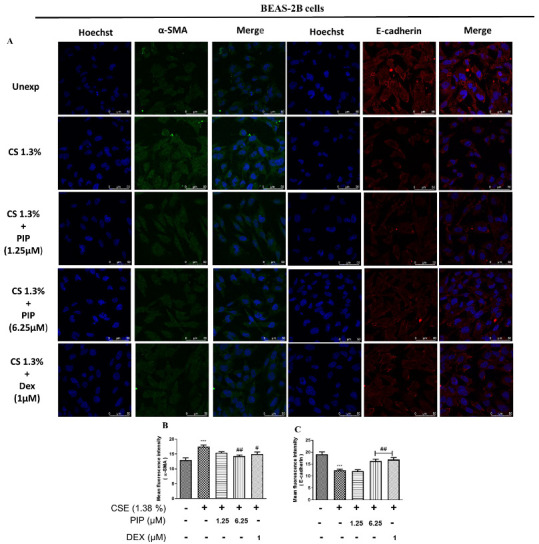
Effect of piperine treatment on expression of EMT signaling in CSE-exposed airway cells. (**A**) Immunofluorescence staining assay in BEAS-2B cells representing an expression of α-SMA, E-cadherin. Bar graphs representing a mean fluorescence intensity of (**B**) α-SMA and (**C**) E-cadherin. The values are expressed as mean ± SEM (*n* = 3). *** *p* < 0.001 vs. Unexposed and # *p* < 0.05, ## *p* < 0.01 vs. CSE. For BEAS-2B cells: CSE: cigarette smoke extract-treated (1.38%), CS + PIP (1.25): cigarette smoke extract + 1.25 µM PIP, CS + PIP (6.25): cigarette smoke extract + 6.25 µM PIP, CS + DEX (1): cigarette smoke extract + 1 µM DEX.

Oxidative stress, inflammation, and Wnt are the primary activators of the EMT process [27]. Persistent inflammation and oxidative stress in airway cells lead to airway remodeling. CS exposure induced increased Vimentin, N-cadherin, α-SMA expression, and decreased E-cadherin expression compared with the unexposed group (Figure 4A–J, *p* < 0.05), indicating a shift towards mesenchymal type due to EMT activation. The PIP (1.25 µM in A549 cells; 6.25 µM in BEAS-2B cells) and standard DEX (1 µM) treatments resulted in an increase in E-cadherin (*p* < 0.01), and a decrease in vimentin (*p* < 0.05), N-cadherin (*p* < 0.01), and α-SMA (*p* < 0.05) compared with the CSE-exposed groups in both cells (Figure 4A–J). Furthermore, immunofluorescence analysis of E-cadherin and Vimentin protein expression in A549 cells also suggests that PIP (1.25 µM) treatment inhibited EMT via upregulating the E-cadherin and downregulating the Vimentin protein expression when compared with the CSE-exposed cells (Supplementary Figure S2A–D, *p* < 0.05). Similarly, in BEAS-2B cells PIP treatment also normalized the EMT changes when compared with the CSE-treated group (Figure 5A–C, *p* < 0.001). Furthermore, immunofluorescence staining of proteins α-SMA and E-cadherin in BEAS-2B cells also confirmed that PIP treatment restored the EMT, similarly to the DEX- (1 µM) treated groups (Figure 5A–C, *p* < 0.01). Overall, it demonstrated that PIP effectively reverses the epithelial to mesenchymal shift due to CS exposure in both lung epithelial cells. To confirm whether the NF-κB and Nrf2 inhibition of PIP (6.25 µM) translated to an inhibition of the EMT process, we estimated the levels of E-cadherin and N-cadherin. We compared the effect of PIP against standard NF-κB and Nrf2 inhibitors, Bay-11 (1 µM) and ML-385 (2 µM), respectively, in CSE- (1.38% *v/v*) exposed BEAS-2B cells (Figure 4F–J, *p* < 0.05). We observed that at a high concentration of PIP (6.25 µM) and with a standard NF-κB inhibitor, Bay-11 (1 µM) treatment increased the epithelial marker E-cadherin and downregulated the mesenchymal marker N-cadherin expressions compared with the CSE-exposed group (Figure 4F–J, *p* < 0.05), indicating the EMT inhibition via NF-κB inhibition. However, the treatment with ML-385 worsened the EMT process as the mesenchymal marker N-cadherin expression remained high and diminished E-cadherin expression, similar to that of the CSE group (Figure 4F–J), suggesting that EMT activation was independent of Nrf2 protein.

### 2.6. Piperine Restores the CS-Induced Altered Lung Mechanics in Mice

**Figure 6:**
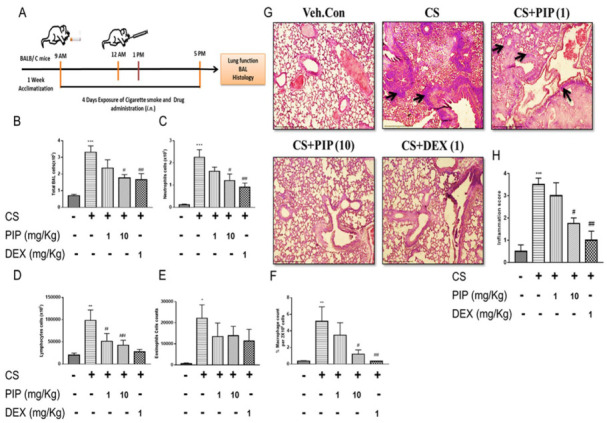
Effect of piperine treatment in an experimental model of smoke-induced inflammation: (**A**) Pictorial representation of in vivo study design. (**B**) Total cell count, (**C**) Neutrophils (**D**) Lymphocytes, (**E**) Eosinophils and (**F**) Macrophages in BAL fluid of various groups. (**G**) Pictorial representation of histopathological analysis (magnification x 100) performed by H and E staining of various groups. → represents influx of cellular infiltration. (**H**). Bar graph depicting inflammation scoring of various groups. The values are expressed as mean ± SEM (*n* = 3). * *p* < 0.05, ** *p* < 0.01 and *** *p* < 0.001 vs. Veh.Control, # *p* < 0.05 and ## *p* < 0.01 vs. CS. Veh.Control: Vehicle control, CS: cigarette smoke treated (9 cigarettes per day for 4 days), CS + PIP (1): cigarette smoke + 1 mg/kg PIP, CS + PIP (10): cigarette smoke + 10 mg/kg PIP, CS + DEX (1): cigarette smoke + 1 mg/kg DEX.

**Figure 7:**
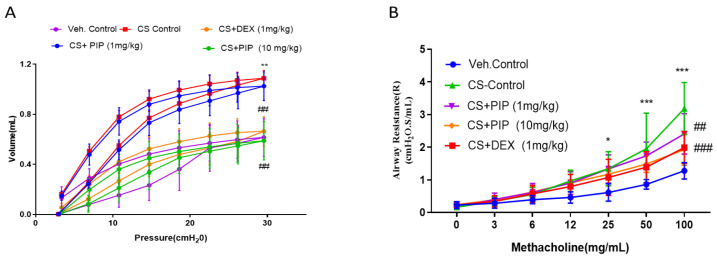
Effect of piperine on CS-induced respiratory functional changes in vivo: (**A**) Mean Pressure-Volume curve at baseline for various groups. Pressure-volume (PV) loops were generated using a ramp-style pressure-volume maneuver (PVr-P). (**B**) Mice were subjected to an increasing dose of nebulized MCh challenge protocol to assess characteristics of airway hyper-responsiveness as central airway resistance (Rn). The values are expressed as mean ± SEM (*n*= 6). * *p* < 0.05, ** *p* < 0.01 and *** *p* < 0.001 vs. Veh.control, ## *p* < 0.01 and ### *p* < 0.001 vs. CS. Veh.control: Vehicle control, CS: cigarette smoke treated (9 cigarettes per day for 4 days), CS + PIP (1): cigarette smoke + 1 mg/kg PIP, CS + PIP (10): cigarette smoke + 10 mg/kg PIP, CS + DEX (1): cigarette smoke + 1 mg/kg DEX.

To further validate the in vitro findings, we used a model of CS-induced inflammation in mice, four days of (3 cigarettes, 3 times per day, Figure 6A), followed by treatment with PIP at doses of 1 mg/kg and 10 mg/kg by the intranasal route (i.n). CS exposure severely affects lung mechanics, such as airway hyperresponsiveness and impaired pressure-volume (PV) loop, due to excessive disruption of normal lung architecture. These parameters are indicative of lung function and its efficacy. We evaluated the mechanical behavior of the chest wall by measuring lung inflation and deflation during the PV curve. CS-exposed animals demonstrated a distinctive upward shift of the PV loop, in which PIP (10 mg/kg) and standard DEX (1 mg/kg) treatment prevented distortion in the PV loop mechanics compared with the CS-group (Figure 7A, *p* < 0.001). Furthermore, we tested the mice with increasing concentrations of methacholine (MCh) in all the groups for changes in airway hyperresponsiveness. We found that airway responsiveness to MCh in the CS-exposed animals was higher, as displayed by the elevation in the airway resistance (Rn) at 25, 50, and 100 mg/mL of MCh when compared with the unexposed animals (Figure 7B, *p* < 0.001). However, PIP treatment (10 mg/kg) significantly reduced CS-induced airway resistance to MCh (at 100 mg/mL MCh, Figure 7B, *p* < 0.01). The PIP treatment of 10 mg/kg was equipotent to the standard DEX (1 mg/kg) treatment to prevent a CS -induced distortion of lung mechanics. 

### 2.7. Piperine Inhibits The Release of Inflammatory Cytokines and Reduces Immune Cells Infiltration in Isolated BAL Fluid and Lung Tissues 

**Figure 8:**
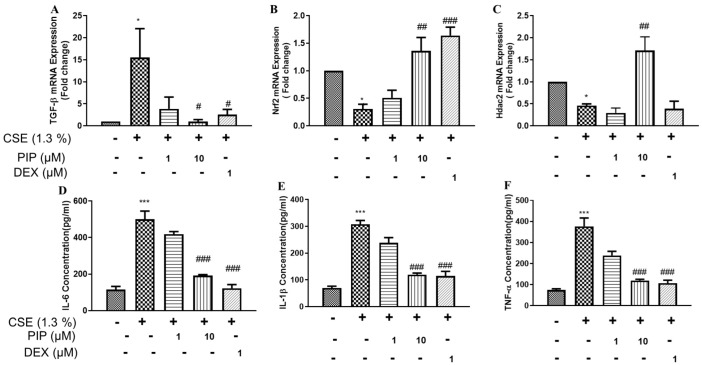
Effect of piperine on oxidative stress and inflammatory marker expression: (**A**) Nrf2 gene, (**B**) TGF-β gene and (**C**) HDAC2 gene in RNA isolated from lung tissue of various groups. Inflammatory markers estimated in BAL fluid of various groups- (**D**) IL-6 expression, (**E**) IL-1β expression and (**F**) TNF-α expression. The values are expressed as mean ± SEM (*n* = 3–6). * *p* < 0.05, *** *p* < 0.001 vs. Veh.Control, # *p* < 0.05, ## *p* < 0.01 and ### *p* < 0.001 vs. CS. Veh.Control: Vehicle control, CS: cigarette smoke treated (9 cigarettes per day for 4 days), CS + PIP (1): cigarette smoke + 1 mg/kg PIP, CS + PIP (10): cigarette smoke + 10 mg/kg PIP, CS + DEX (1): cigarette smoke + 1 mg/kg DEX.

We found that CS exposure leads to impaired lung function in animals. To validate the in vivo findings, we measured the differential cell count in BAL and performed lung histology (H&E stain). CS exposure resulted in immune cell influx in the airways as measured in BAL compared with unexposed mice (Figure 6B, *p* < 0.001). Significant increases in neutrophils (Figure 7C, *p* < 0.001), lymphocytes (Figure 6D, *p* < 0.001), and macrophages (Figure 6E, *p* < 0.05) were observed in the BAL fluid, lung tissue histology, and inflammation score (Figure 6G, H, *p* < 0.001) of CS-exposed animals when compared with veh. control animals. Interestingly, PIP treatment at a 10 mg/kg dose significantly reduced the CS-induced influx of immune cells in the BAL (Figure 6B–F, (*p* < 0.01) and lung histology, with an inflammation score (Figure 6G,H, *p* < 0.05) equivalent to that of the DEX treated group (1 mg/kg). Furthermore, PIP treatment at 10 mg/kg showed a reduction in various cytokines IL-1β, TNF-α, and IL-6 compared with the CS-exposed group, similar to the DEX treated group (Figure 8D–F, *p* < 0.001). Our data revealed that PIP treatment decreases inflammation by decreasing the secretion of inflammatory cytokines.

### 2.8. Piperine Curtails CS-Induced Oxidative Stress and Inflammatory Signaling in Isolated Lung Tissues

Lipid peroxidation (malondialdehyde, MDA), Glutathione (GSH), and nitrite levels (NO) are critical markers for the assessment of CS-induced oxidative stress. In the lung lysates of CS-exposed animals, MDA (Supplementary Figure S3A, *p* < 0.05) and Nitrite (Supplementary Figure S3C, *p* < 0.05) levels were significantly elevated, while levels of GSH (Supplementary Figure S3B, *p* < 0.01) were reduced. MDA levels are normalized upon PIP treatment (10 mg/kg, Supplementary Figure S3A, *p* < 0.01) compared with the CS-exposure group. However, PIP treatment (10 mg/kg) was able to restore GSH and NO levels compared with the CS-exposure group alone (Supplementary Figure S3B,C, *p*< 0.05). Interestingly, animals treated with DEX (1 mg/kg) failed to restore the significant changes in GSH and NO levels induced by the CS-exposed group. CS-induced dysregulation of pro-oxidant-antioxidant balance and inflammation in the lung was further confirmed by a qPCR gene expression analysis. CS significantly induced TGF-β mRNA expression, which was abrogated entirely with PIP treatment (10 mg/mL, Figure 8A, *p* < 0.05), equivalent to the DEX (Figure 8A, *p* < 0.05) treatment. CSE exposure also resulted in decreased mRNA expression levels of lung antioxidant markers, Nrf2, which were restored upon treatment with PIP (10 mg/kg, Figure 8B, *p* < 0.01) and DEX (1 mg/kg, Figure 8B, *p* < 0.001) equally. Fascinatingly, PIP treatment showed upregulation in the mRNA expression of HDAC2 compared with CSE-exposed animals (Figure 8C, *p* < 0.01). In contrast, DEX treatment displayed downregulation of HDAC2, similar to that of CS-exposed animals (Figure 8C, *p* < 0.05). Our results demonstrated that PIP has the potential for the reversal of corticosteroid resistance by promoting HDAC2, an essential component for the pharmacological action of corticosteroids.

### 2.9. Piperine Inhibits EMT Changes and Modulates SIRT1 Signaling in Isolated Lung Tissues 

**Figure 9:**
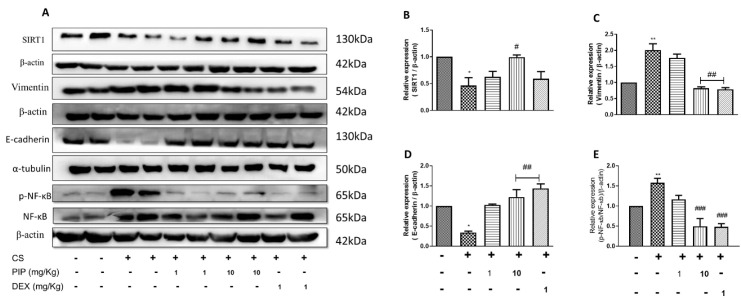
Effect of piperine treatment on expression of EMT changes, antioxidant, and SIRT1 signaling in CS-exposed murine model: (**A**) Immunoblot images representing the expressions of SIRT1, E-cadherin, N-cadherin, Vimentin, and p-NF-κB/NF-κB. Bar graphs representing densitometry analysis of (**B**) SIRT1 (**C**) vimentin, (**D**) E-cadherin and (**E**) p-NF-κB/NF-κB. The values are expressed as mean ± SEM (*n* = 3). * *p* < 0.05 and ** *p* < 0.01 vs. Unexposed, # *p* < 0.05, ## *p* < 0.01 and ### *p* < 0.001 vs. CS. CS: cigarette smoke exposure, CS + PIP (1): cigarette smoke exposure + 1 mg/kg PIP, CS + PIP (10): CS + 10 mg/kg PIP, CS + DEX (1): CS + 1 mg/kg DEX.

The CS-exposed group showed a reduction in the expression of SIRT1, E-cadherin, and upregulation of Vimentin and p-NF-κB compared with the veh. control (Figure 9A–E, *p* < 0.05). The results are similar to in vitro data. Animals exposed to CS along with the treatment of PIP (10 mg/kg) displayed restoration of E-cadherin expression levels and decreased Vimentin and p-NF-κB when compared with the CS-exposed group (Figure 9A–E, *p* < 0.01), similar to the DEX-treated animals. Interestingly, treatment with PIP (10 mg/kg) displayed normalization of SIRT1 expression levels, whereas the DEX-treated group was unable to do so compared with CSE-exposed animals (Figure 9A,B, *p* < 0.05). Our data indicate that PIP can inhibit EMT by enhancing the SIRT1 signaling.

## 3. Discussion

The novel findings of the current study are that: (a) PIP protects against CSE-induced epithelial cell death, (b) PIP significantly normalizes the epithelial markers and downregulates the mesenchymal markers using in vivo and in vitro models against CS exposure. (c) PIP attenuates CS-induced oxidative stress, inflammation, EMT, and conserves lung function by inhibiting oxidative stress and enhancing SIRT1 signaling (d) PIP upregulates the HDAC2 gene expression and might reverse the corticoid resistance induced by CS.

The recent coronavirus (COVID-19) pandemic has highlighted the lack of protective pulmonary-anti-inflammatory agents that could be used long-term to reverse the pulmonary remodeling due to inflammation. The lack of pulmonary protective agents poses the risk of developing long-term pulmonary and extrapulmonary complications. Recent analysis of clinical studies by WHO concluded that both active and passive smoking could aggravate coronavirus severity [28]. The large-scale world-wide vaccination drives were able to curb COVID-19 mortality. However, the sub-infectious levels of native and mutated strains of SARS-CoV-2 (Coronavirus) will continue to cause pulmonary and extrapulmonary complications. The CS exposure and sub-infectious levels of coronavirus strains will exacerbate the long-term effects of coronavirus-induced-pulmonary and extrapulmonary complications [29,30]. Clinically, anti-inflammatory and bronchodilator agents such as corticosteroids and salbutamol are used to ameliorate pulmonary inflammation. These drugs have little to negligible impact on the cellular epigenetics that are altered due to sustained inflammation [31]. 

Cigarette smoke is one of the well-known risk factors for causing chronic lung diseases by activating oxidative stress–inflammation-mediated signaling cascades and ultimately leading to airflow limitation and small airway fibrosis [32]. Emerging studies provide evidence that the epithelial–mesenchymal transition (EMT) and oxidative stress are the critical mediators that get activated in chronic lung diseases and are responsible for its progressive nature. However, targeting the EMT and oxidative stress can be an effective and novel way to minimize small airway annihilation [33]. Several reports have been published suggesting the role of PIP due to its anti-inflammatory, antioxidant, and antifibrotic properties [13,15,21]. However, its therapeutic potential on CS-induced pulmonary inflammation, SIRT1, and EMT signaling remains unexplored. Corticosteroids stand as the first line of therapy in chronic airway disease, primarily COPD and asthma. However, long-term use may result in corticosteroid resistance and serious unwanted effects. Therefore, an attempt has been made to minimize the use of corticosteroids, which not only prevents resistance development but also provides more efficient management of the disease and ensures patients’ safety by avoiding steroid-associated adverse effects. Using a murine model, our study evaluated the effects of intranasally administered PIP compared with DEX (corticosteroid) on CS-induced airway inflammation, EMT, SIRT1 signaling, oxidative stress, and lung function. Additionally, the cellular mechanisms by which PIP may exert its therapeutic benefits were explored in human alveolar epithelial (A549) cells and bronchial epithelial cells (BEAS-2B) in in vitro models.

Chronic inflammation in COPD and asthma, triggered by repeated exposure to harmful particles/gases such as cigarette smoke and allergens, results in airway remodeling. Corticosteroids possess anti-inflammatory properties by inhibiting the histone acetylation of inflammatory genes when binding with glucocorticoid receptors [34]. Evidence suggests that significant drawbacks of corticosteroids are steroid resistance, pneumonia, and death [35]. Steroid resistance occurs due to oxidative stress/nitrosative stress, which significantly decreases the HDAC2 levels that are essential for steroid action [34]. Drugs such as PDE-4 inhibitors (Roflumilast), Adenosine receptor inhibitors, and cytokine inhibitors were limited due to their side effects, lack of efficacy, resistance, clinical failure, and increased patient withdrawal from clinical studies [36]. Thus, alternative therapeutic strategies are needed to overcome the current therapy’s limitations.

Piperine (PIP) is one of the active ingredients in the long and black peppers. It has been widely used, traditionally and in various ayurvedic formulations, for treating sore throats and coughs [37]. Jun Soo Bang et. al, reported that PIP demonstrates anti-inflammatory properties at a dose of 100 mg/kg/day per oral in the arthritis model [16]. Multiple studies have demonstrated the anti-inflammatory action of PIP by modulating NF-κB and Nrf2. PIP also demonstrated in vitro anti-inflammatory action by inhibiting NF-κB in LPS-induced inflammation in RAW 264.7 cells (in murine macrophage cell line) [38]. In another study, PIP treatment prevented colon cancer by inducing Nrf2 antioxidants and inhibiting the NF-κB pathway in 1,2–Dimethylhydrazine-induced colon cancer [39]. Furthermore, PIP also inhibits TGF-β and EMT in A549 which led us to speculate that PIP might dampen the CS-induced airway inflammation and EMT-related signaling [18]. Based on the Jun Soo Bang et al. report, we selected 10-fold reduced doses, i.e., a high dose (10 mg/kg/day) and a low dose (1mg/kg/day), administered intranasally at a dose volume of 50 µL in CS-induced airway inflammation using a mouse model.

The current study explores the lung protective properties of PIP against CS-induced pulmonary inflammation and its related signaling in vitro and in vivo. The in vitro model exposes CSE in A549 (alveolar epithelial) and BEAS-2B (Bronchial epithelial) cells to simulate airway inflammation, EMT, and related signaling. The CSE was prepared by bubbling cigarettes in PBS. Nicotine, a principal water-soluble constituent of CS responsible for toxic and addictive effects, was estimated in prepared extracts. We observed that CSE had a nicotine concentration of 367 ng/mL. Therefore, 48 hrs exposure of 3% and 1.38% of CS extract was used for simulating A549 and BEAS-2B, containing approximately 11.01 ng/mL and 5.06 ng/mL nicotine concentrations, respectively (Supplementary Figure S1). The anti-inflammatory effects of PIP were equipotent to DEX. PIP’s antioxidant and anti-inflammatory effect was due to the modulation of a SIRT-1 and Wnt-mediated signaling cascade. PIP upregulated SIRT-1, Nrf2 and downregulated NF-κB and β-catenin to restore oxidative balance, inhibit inflammation, and EMT. In order to confirm whether the NF-κB and Nrf2 inhibition modulated the SIRT1, EMT, and inflammation, the CSE-exposed A549 and BEAS-2B cells were treated with Bay-11 (NF-κB inhibitor) and ML-385 (Nrf2 inhibitor). The NF-kB inhibition resulted in upregulation of E-cadherin and SIRT-1 and inhibition of N-cadherin and α-SMA, indicating inhibition of oxidative stress, inflammation, and EMT [40]. The effects were similar to the PIP treatment, which inhibited N-Cadherin and α-SMA, and upregulated SIRT-1 and E-cadherin. The higher concentration of PIP showed consistent results of effectively inhibiting oxidative stress, inflammation, and EMT. However, ML-385 (Nrf2 inhibitor) worsened the disease condition and promoted the EMT transition. The exacerbation of EMT by ML-385 (Nrf-2 inhibitor) indicates that oxidative stress and inflammation need to be inhibited by reversing cigarette smoke-induced-epigenetic changes and airway remodeling by upregulating SIRT-1 and E-cadherin and inhibiting N-cadherin and α-SMA.

Airway hyperresponsiveness (AHR) is a key indicator of lung mechanics used for detecting airway resistance, airflow, and respiratory rate, and can also be used to assess pulmonary function in patients with chronic airway diseases. In chronic airway disease, persistent airway inflammation leads to a gradual drop in lung function, further resulting in airway hyperactivity, airflow limitation, and breathlessness. In our study, we measured respiratory mechanics to assess CS-induced impairment of airway responsiveness at both baseline and with increasing concentrations of methacholine. Several studies have shown increased bronchial sensitivity to MCh after smoking [41]. Our study confirmed the impairment of lung function as seen with an increase in conducting airway resistance along with an upward shift of the PV loop upon CS exposure. As it is known that structural changes in the lung can result in functional deficits independent of inflammation [42], we found that CS exposure can lead to an alteration of lung structure and function, leading to airflow limitation. PIP treatment at a dose of 10 mg/kg has the potential to reduce airflow limitation leading to a restoration of impaired lung function in CS-induced altered respiratory function in mice. 

Inflammation persists in chronic airway disease patients even after smoking cessation due to the regulation of crucial inflammatory cell types, such as macrophages and lung epithelial cells, which produce various inflammatory mediators, such as TGF-β, IL-1β, and TNF-α [43]. The regulation of these cytokine productions is led by NF-κB [44]. The present study has shown the increased expression of p-NF-κB in vivo upon CS exposure (9 cigarettes per day for 4 days). Treatment with PIP (10 mg/kg) and DEX (1 mg/Kg) inhibited the activation of the translocation of phosphorylated NF-κB into the nucleus. Hence, there was a decrease in the production of inflammatory mediators in CS-exposed mice. These results confirm the anti-inflammatory role of PIP which has previously been shown in inflammatory models [14]. Cigarette smoking causes immune cell migration into the lungs of COPD patients [45]. Clinical and animal studies have confirmed these findings with blood and BALF differential cell counts. Thus, in the present study, we have confirmed inflammation’s significance with BAL cytology and lung histopathological analysis. These findings have shown an increased total cell count, including macrophages, lymphocytes, and neutrophils in BALF and the deposition of neutrophils around the peribronchial lineage on CS insult. However, PIP treatment (10 mg/Kg) and DEX (1 mg/Kg) treatment have significantly reduced the BAL cell count, accumulation of neutrophil cells in the lung, and decreased cytokine production in BAL fluid. Furthermore, our ELISA analysis of secretory cytokines such as TNF-α, IL-1β, and IL-6 in BALF also confirms that PIP (10 mg/Kg) and DEX (1 mg/Kg) treatment significantly decreased the cytokines levels, which indicates that PIP may show anti-inflammatory properties by reducing the release of cytokines.

Cigarette smoking is well known for the induction of oxidative stress, which increases cellular oxidants and causes inflammation [46]. Cigarette smoke generates more than 1017 free radicals, contributing to the primary cause of increased oxidative stress in COPD patients [47]. The free radicals play a crucial role in activating inflammatory signals in epithelial cells and inducing oxidative stress, leading to an enhanced lipid peroxidation rate and pro-inflammatory cytokine production via NF-κB activation [48]. Recent reports also evidence that CSE increases ROS due to the inhibition of NRF2/KEAP1 pathways [49]. Thus, targeting oxidative stress via an enhancement of antioxidant mechanisms in COPD could be salutary in diminishing persistent lung damage that progresses over time, leading to disease aggravation. However, in clinical trials, antioxidants molecules such as NAC (N-acetyl cysteine) and carbocystein failed to establish efficacy in COPD [50]. In line with the previous study, our experiments confirm that PIP reduced oxidative stress by upregulating the antioxidant mechanism by activating the Nrf2 signaling.

Oxidative stress and inflammatory cytokines generation may be detrimental to the airway walls making them more susceptible to an alteration in structure and function. Airway remodeling, a key feature observed in COPD and asthma, can be linked with the activation of EMT signaling [51]. In the present scenario, no therapy could target EMT with anti-inflammatory and enhance the antioxidant mechanism in COPD patients. Airway epithelial cell remodeling results from direct exposure to cigarette smoke or activation of inflammatory mediators such as TGF-β, TNF-α, etc. [52] Moreover, PIP has previously been established as having anti-inflammatory properties [14]. Consistent with previous reports, our data also showed that PIP blocked the EMT by reducing the expression of mesenchymal markers Vimentin, α-SMA, and N-cadherin and restoring the epithelial marker E-cadherin. EMT is regulated by several interlinked downstream signaling cascades such as the Wnt pathway which is implicated in lung damage [53]. Our findings suggest that CS downregulates p-β-catenin thereby preventing β-catenin degradation resulting in β-catenin translocation into the nucleus and consequently affecting various downstream signaling that affects EMT and inflammation. PIP treatment at a dose of 10 mg/kg blocked the EMT signaling in a similar way to standard DEX (1 mg/kg) due to its anti-inflammatory and antioxidant mechanism.

We further explored the mechanisms through which PIP protects CSE-induced EMT and inflammation. SIRT1, as a histone deacetylase enzyme, plays a pivotal role in CS-induced airway remodeling response [54]. Several reports have demonstrated the reduced expression of SIRT1 in the lungs of CS-exposed mice and in smokers and COPD patients [55]. We found that PIP treatment alleviated CSE-induced downregulation of SIRT1 expression. Previous reports demonstrated that SIRT1 was downregulated in bleomycin (BLM)-induced pulmonary fibrosis, and treatment with an SIRT1 activator attenuated the EMT in BLM-induced fibrosis [56]. Reports also suggested that Hydrogen sulfide alleviates CS-induced airway remodeling via an enhancement of the SIRT1 signaling [56]. In agreement with previous reports, our data confirms that PIP protects the CSE-induced oxidative stress, EMT, and inflammation by upregulating the SIRT1 signaling pathway.

Several reports suggested that corticoid resistance occurs due to oxidative stress/peroxynitrites, which subsequently diminishes the HDAC2 protein, which plays a crucial role in corticoid pharmacological action [34]. Recent reports demonstrated that andrographolide overcomes the corticosteroid resistance in COPD by modulating the HDAC2 and Nrf2 expression [57]. Consistent with previous reports, PIP treatment in mice displayed upregulation of HDAC2 mRNA expression. Thus, PIP might be able to reverse the corticoid resistance via modulation of HDAC2 and Nrf2 protein expressions. Our study has a limitation, i.e., the use of an acute CSE-exposure murine model as this does not fully mimic the lung functional impairments observed in COPD and asthma patients. We proposed that an increase in MCh responsiveness in this model is likely a result of the magnified CSE-induced acute inflammation, impairment of antioxidant response in the lung, and activation of EMT-related signaling. However, further studies are required to see the effect of PIP in combination with steroids to prevent resistance.

## 4. Materials and Methods

### 4.1. Materials

Piperine (PIP) (purity ≥ 97%) was purchased from Sigma-Aldrich having cat no- P49007-1G. For cell culture experiments, PIP was dissolved in molecular grade dimethylsulfoxide (DMSO) in a stock solution of 20 mM. For mouse experiments, PIP was freshly formulated as 60 mg/mL and 6 mg/mL in vehicle (0.2% DMSO + one drop tween-80 in sterile PBS). All the other chemicals used are of an analytical grade and were purchased from Sigma Aldrich (USA), Thermo Scientific (USA) etc.

### 4.2. Preparation of Aqueous Cigarette Smoke Extract (CSE)

Cigarette (Marlboro Red) smoke was bubbled into PBS and filtered through a sterile 0.2 µM membrane filter. The collected filtrate was considered 100% and then diluted with media to prepare different concentrations of CSE. CSE was used to expose A549 and BEAS-2B cells for dose estimation, cellular toxicity assessment, staining, molecular analysis, etc.

### 4.3. Cell Viability Assay

A549 cells (Human lung epithelial cells) and BEAS-2B cells (Human bronchial epithelial cells) were seeded in 96 well plates containing respective media supplemented with 10% FBS. Cells were allowed to attain morphology followed by serum deprivation and then exposed to different concentrations of CSE, PIP, and Dexamethasone (DEX 1 µM), a standard drug. Cellular toxicity assay was determined using Thiazolyl Blue Tetrazolium Bromide (MTT, Sigma Aldrich, USA). After 4 h, MTT was discarded, DMSO (Sigma Aldrich) was added, incubated, and absorbance was measured at 570 nm. IC_50_ of CSE and and EC_90_ of PIP were calculated, respectively. A further protective assay was performed using a 5-fold diluted concentration of EC90 of PIP as a high dose and a 5-fold diluted concentration as a low dose for post 1 h treatment after the IC50 of CSE exposure. 

### 4.4. Immunofluorescence

Cells were exposed with IC50 of CSE followed by post 1 hr treatment with PIP at a concentration of 6.25,1.25, and 0.25 µM, and DEX 1 µM. After 48 h, cells were washed and fixed with 4% paraformaldehyde. Cells were permeabilized using 0.3% Triton X-100, then blocked for 1 h followed by overnight incubation with a primary antibody (E-cadherin, 1:300, α-SMA 1:300, p-NF-κB 1:300) at 4 °C. Subsequently, cells were washed and then incubated with fluorophore-conjugated secondary antibodies. Further washed and mounted with Prolong Antifade with DAPI (Invitrogen, USA). Image acquisition was made using a confocal laser scanning microscope (CLSM, Leica, Germany). Data were analyzed using Leica Las-X software in terms of mean fluorescence intensity. 

### 4.5. Cigarette Smoke Exposure in a Mouse Model

Experiments were performed per the guidelines of the Institutional Animal Ethical Committee (IAEC number- NIPER/PC/19/034). In total, 36 BALB/c mice were used for the study and divided into 5 groups. Veh. control animals were exposed to atmospheric air. In contrast, cigarette smoke groups (±drugs) were exposed to 9 cigarettes per day for 4 days (Figure 4A). PIP and DEX treatments were administered 1 h before the exposure. The treatment groups were Veh. control (0.2% DMSO+ one drop tween-80 in sterile PBS), PIP (1 mg and 10 mg/kg), and standard drug DEX (1 mg/kg), administered by intranasal route daily. Mice were placed inside an 18-L Perspex chamber under the fume hood and exposed to cigarette smoke (CS) generated from 3 cigarettes at the time, thrice a day, for 4 days (Day 0–3). Commercially available Marlboro cigarettes were used for the experiment. After treatment, animals were anesthetized, and lung functions were assessed using Flexivent (Scireq, Canada). Bronchoalveolar lavage (BAL) fluid was collected, followed by lung isolation for biochemical assays, histopathology, and molecular analysis. 

### 4.6. Assessment of Lung Function 

Lung function measurements were performed using a FlexiVent system (Scireq, Canada) [14]. After the final CS exposure, individual mice were anesthetized with Xylazine and Ketamine (10 mg/kg and 100 mg/kg; i.p), followed by intubation using a cannula connected to the ventilation instrument with a breathing rate of 150 breaths/min. Readings were recorded at baseline such as airway resistance (Rn) and Pressure volume curve (PV-Loop). Airway resistance Rn after the nebulization of methacholine (MCh) to determine the airway hyper-responsiveness features. Changes in lung resistance (Rn) were recorded at each dose of MCh 0, 3, 6, 12, 25, 50 and 100 mg/mL.

### 4.7. Assessment of BAL Cytology

BAL fluid was collected and subjected to centrifugation to separate the cells. The supernatant was stored at −80 °C, and a cell pellet was used for differential cell counts (neutrophils, lymphocytes, eosinophils) as well as total leukocyte counts using an automatic hematological analyzer (Siemens Advia 2120i analyzer, Germany), while based on surface markers like CD64, Siglec F, CD11b and F4/80, macrophage counts were recorded using flow cytometry (Attune-NxT, Thermo Scientific, USA) [15].

### 4.8. Measurement of Biochemical Parameters 

Lung tissue homogenates were used to estimate Lipid peroxidation, Nitrite level, and Reduced Glutathione. Malondialdehyde (MDA) levels were measured to assess the extent of lipid peroxidation using thiobarbituric acid (TBA). Reduced glutathione (GSH) levels were estimated by using Ellman’s method. Griess reagent was used to estimate Nitrite levels according to our optimized lab protocol [23]. 

### 4.9. RNA Isolation and Real-Time Polymerase Chain Reaction

Total RNA was isolated from the lung tissue using Trizol reagent (Invitrogen). After isolation, cDNA was prepared using the cDNA Synthesis Kit (Takara Bio, Japan) per manufacturer protocol. Sybergreen master mix (Invitrogen) was used to perform quantitative PCR. (Applied Biosystems Real-time PCR machine, Quantstudio-5). GAPDH was used as a housekeeping gene to calculate gene expression [23]. Nrf-2 (F-5′ CAGCATGTTACGTGATGAGG 3′, R-5′ GCTCAGAAAAGGCTCCATCC 3′), TGF-β (F-5′ CGCAACAACGCCATCTATGA 3′, R-5′ GGCTGATCCCGTTGATTTCC 3′), HDAC-2 (F-5′ TGCAGAGATTTAACGTCGGA 3′), (R-5′ TGGCATGATGTAGTCCTCCA 3′) and GAPDH (F-5′ CTGCCCAGAACATCATCCCT 3′), (R-5′ TCATACTTGGCAGGTTTCTCC 3′).

### 4.10. Histopathological Analysis 

The inflated left lung was used for a histopathology study performed as described previously [16]. Lung tissue was processed in a sequential series of alcohol followed by xylene and then, lastly, paraffin infiltration and embedding. Sections were cut in a microtome, mounted on a slide, and kept dry. Once dry, they were used for standard staining procedure. The lung sections were deparaffinized in 3 changes of xylene followed by a sequence of graded alcohol and distilled water for rehydration. The slides were stained with haematoxylin for 1 min, washed under tap water, dipped in ammonia solution for 30 s, followed by eosin staining for 30 s, and then washed again. Then sections were dehydrated using absolute alcohol and xylene. Finally, the sections were mounted using DPX reagent. Images were acquired using a Leica microscope (bright field mode).

### 4.11. Western Blotting

Treated A549 and BEAS-2B cells were used for protein extraction. The respective lung tissue homogenate was used for protein extraction. Proteins were separated by using SDS-PAGE. Proteins separated according to molecular weight were transferred to a Nitrocellulose membrane and then blocked with 3% BSA for 1 h. After washing, membranes were incubated with primary antibody E-cadherin, N-cadherin, Vimentin, α-SMA, NF-κB, p-NF-κB, Nrf2, β-Catenin, p-β-Catenin, SIRT1, α-tubulin, and β-actin (1:1000) at 4 °C overnight followed by washing and incubation with HRP linked secondary antibody. Bands were visualized using ECL reagent (Biorad) under Chemidoc Fusion-FX (Vilber Loumart). Image-J software was used for the quantification of visualized bands.

### 4.12. Statistical Analysis

The data were expressed in terms of mean ± SEM. All statistical comparisons were made by two/one-way analysis of variance (ANOVA) followed by post hoc Dunnett’s and Bonferroni’s multiple comparison tests using the graph pad prism version 7.0 software. *p* values *<* 0.05 were considered statistically significant.

## 5. Conclusions

Our study delineates the exact molecular mechanism behind the anti-inflammatory action of PIP in CS-induced lung inflammation in pulmonary epithelial cells. The PIP treatment ameliorates pulmonary inflammation by activating SIRT1-mediated cellular defense against oxidative stress and inflammation. PIP treatment curtailed oxidative stress, inflammation, and EMT by promoting SIRT1 and Nrf2 and inhibiting NF-κB. Thus, PIP may be considered as a potential natural compound for the treatment of oxidative stress, pulmonary inflammation, and EMT-related pathological signaling. PIP also has the potential to reverse CS-induced epigenetic modification, which is crucial to overcoming late-stage corticosteroid resistance. Further pre-clinical toxicity studies and clinical validations need to be explored for its clinical use. 

## Data Availability

The data presented in this study are available on request from the corresponding author.

## Supplementary Materials

The supporting information can be downloaded at: https://www.mdpi.com/article/10.3390/ijms232314722/s1.
