# Supplementary material for: Piperine Attenuates Cigarette Smoke-Induced Oxidative Stress, Lung Inflammation, and Epithelial–Mesenchymal Transition by Modulating the SIRT1/Nrf2 Axis
Source: Int J Mol Sci. 2022 Nov 25;23(23):14722. doi: 10.3390/ijms232314722 (PMC9740588; doi:10.3390/ijms232314722)
Supplement: Supplementary file 1 [file ijms-23-14722-s001.zip › ijms-1936258-supplementary.pdf]

<u>Supplementary Information's</u>

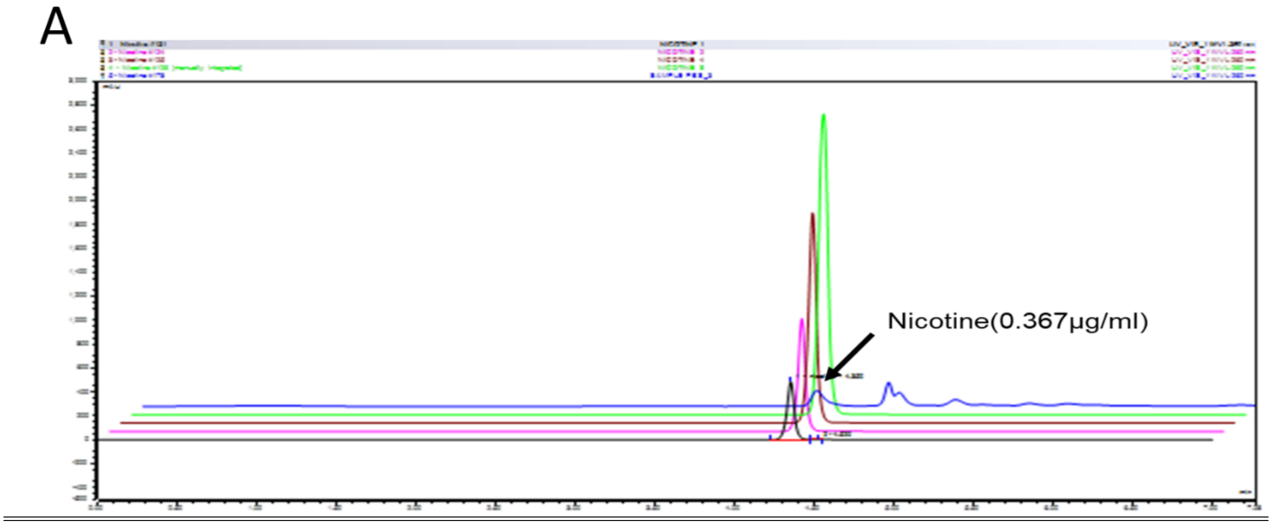

Supplementary Figure S1: Nicotine levels estimation in cigarette smoke extract (CSE) A) Representative HPLC chromatogram overlay of nicotine content in CSE along with the standard concentration for nicotine(n=3). The concentration of nicotine extracted in phosphate buffer saline was estimated by using Dionex Ultimate 3000 UHPLC system equipped with (photodiode array) PDA detector. The chromatographic analysis was performed on Hypersil Gold C8 column (150 X 4.6mm, 5μm) using 0.1% TFA in water and acetonitrile (9:1) as mobile phase with a flow rate of 0.5mL/min. The detection wavelength was 260nm.Nicotine concentration was found to be 0.367μg/ml.

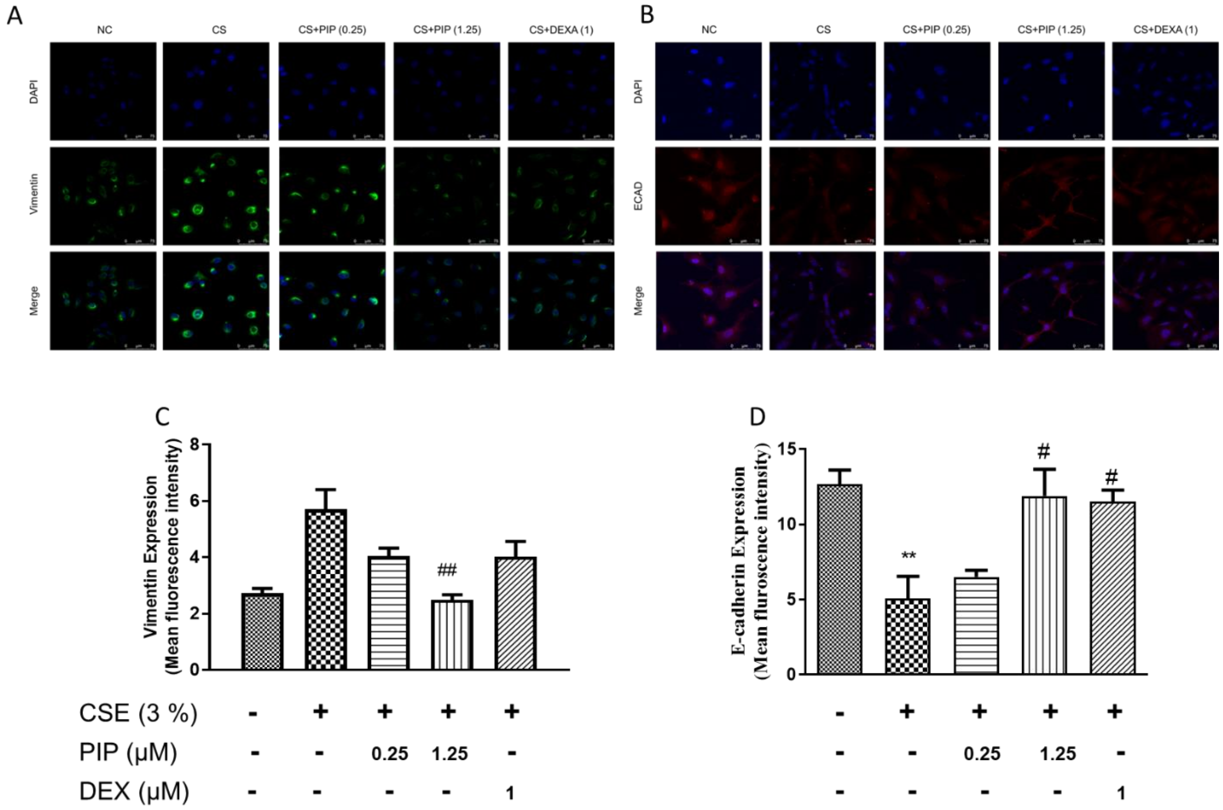

Supplementary Figure S2: Immunofluorescence staining assays represent an expression of (A) Vimentin and (B) E-cadherin in A549 cells. Bar graphs represented a mean fluorescence intensity of (C) Vimentin and (D) E-cadherin. The values are expressed as Mean ± SEM (n=4). ***p*<0.01 vs unexposed, #*p*<0.05 and ##*p*<0.01 vs CSE. CS: cigarette smoke extract-treated (3 %), CS+PIP (0.25): cigarette smoke extract + 0.25µM PIP, CS+PIP (1.25): cigarette smoke extract + 1.25µM PIP, CS+DEX (1): cigarette smoke extract + 1µM DEX.

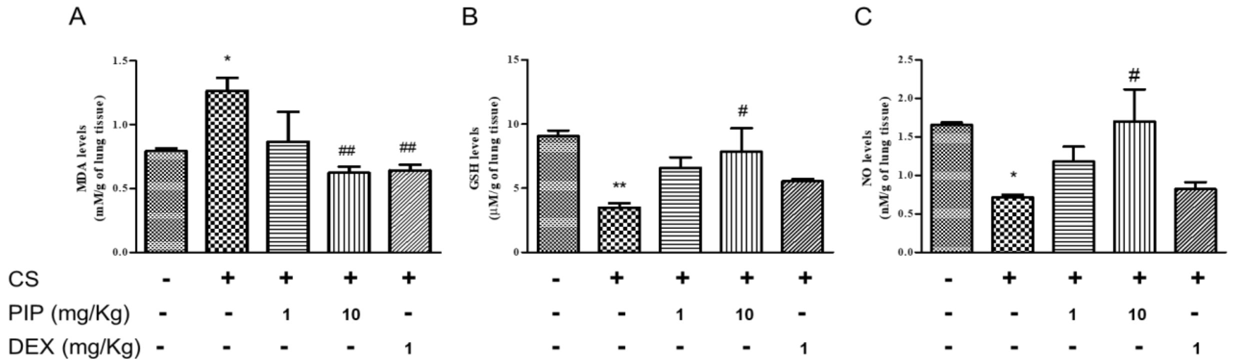

Supplementary Figure S3: Effect of piperine on oxidative stress and inflammatory changes in mice. (A) MDA levels, (B) GSH levels, and (C) Nitrite levels (NO) levels in lung tissue lysates of various groups. The values are expressed as Mean ± SEM (n=6). **p*<0.05 and ***p*<0.01 *vs Veh.Con*, #*p<0.05* and ##*p*<0.01 *vs CS*. Veh.con: Vehicle control, CS: cigarette smoke treated (9 cigarettes per day for 4 days), CS+PIP (1): cigarette smoke + 1 mg/kg PIP, CS+PIP (10): cigarette smoke + 10 mg/kg PIP, CS+DEX (1): cigarette smoke + 1 mg/kg DEX.
